# Childhood Trauma, Recent Stressors, and Risk of Depression Relapse in Late‐Life Depression

**DOI:** 10.1155/da/1455337

**Published:** 2026-10-08

**Authors:** David C. Steffens, Rong Wu, Julian D. Ford

**Affiliations:** ^1^ Department of Psychiatry (DCS, JDF) and Biostatistics Center The Cato T. Laurencin Institute for Regenerative Engineering (RW) University of Connecticut School of Medicine Farmington Connecticut USA uchc.edu

## Abstract

**Background:**

Recent stressors have been linked to poor outcomes of late‐life depression (LLD), including relapse. Emerging evidence has linked adverse childhood experiences (ACEs) to the occurrence of LLD. We sought to examine the relationships between recent exposure to stressful life events and ACEs with relapse following remission among older adult.

**Methods:**

In this cohort study, depressed older adults (age ≥65) were provided standard antidepressant medications by a study psychiatrist. Each participant provided responses to the ACEs Questionnaire and to a list of questions regarding stressors in the past 12 months. We defined remission as Montgomery–Asberg Depression Rating Scale (MADRS) score <8 and relapse worsening with a MADRS score ≥15. We conducted both logistic regression analyses of relapse over 3 years and a time‐to‐relapse survival analysis. To assess moderation by ACE items on the association of stress and relapse, we included them in Cox proportional hazard (PH) models.

**Results:**

Of 143 depressed elders, 98 achieved remission within 2 years, and 55 of them experienced a relapse. Exposure to emotional neglect in childhood and stressors later in life were each associated with relapse of LLD, and a history of childhood neglect moderated the relationship between recent stressor exposure and relapse. In bivariate analyses, ACE item 4 (emotional neglect), ACE item 9 (family mental illness), and the combination of both (ACE4 + ACE9) were significantly related to relapse. In subsequent logistic regression models controlling for age, sex, race, education, and baseline MADRS score, the dichotomous and continuous variables combining ACE items 4 and 9 were significantly associated with relapse. In analyses using time‐to‐relapse as the outcome, emotional neglect was associated with a shorter time to relapse. The number of recent negative stressors was also associated with relapse, and emotional maltreatment moderated that relationship in an unexpected way.

**Conclusion:**

We found that exposure to emotional neglect in childhood and stressors later in life both were associated with relapse of LLD, and a history of childhood neglect moderated the relationship between recent stressor exposure and relapse. These findings are consistent with, and extend, the robust research evidence base linking psychological neglect with depression in adolescence and adulthood.

## 1. Introduction

Late‐life depression (LLD) occurs in up to 5% of the elderly, is associated with increased suicide risk, aggravates existing medical conditions, increases functional disability, and negatively impacts cognition [1, 2]. A previous national representative sample found that, in the U.S., the combined prevalence of minor and major depression is 11% [3]. LLD increases the risk for dementia, both Alzheimer’s disease (AD) [4–6] and vascular dementia [7]. While antidepressant medications and psychotherapy can effectively treat depression in older adults, those who respond remain at high risk for future episodes [8]. In well‐controlled clinical trials, high recurrence rates are reported, with about 35% of elderly receiving antidepressants experiencing a recurrence over 2 years [9, 10]. In a naturalistic cohort study, LLD participants receiving antidepressant treatment had recurrence rates of 43% over 2 years and 57% over 4 years [11].

There is a growing emphasis on stress and the poor outcomes of depression. Recurrent depression in nongeriatric adults is associated with altered physiologic stress reactivity to both mild and more potent stressors, e.g., activation of the hypothalamic–pituitary–adrenal (HPA) axis and the sympathetic nervous system [12–15]. Such stress‐related physiological responses may predict subsequent recurrent depressive episodes [16]. In LLD, higher perceived stress was associated with an increased risk of recurrence [11]. Recurrence risk has also been associated with more negative stressful life events in LLD [17, 18]. However, in another study, life stressors occurring later in life were not associated with relapse in LLD [19].

While relapse and recurrence each refer to a return of symptoms consistent with a depressive episode, there are recommended timelines for considering relapse vs. recurrence in depressed adults. For example, in 2006, the American College of Neuropsychopharmacology (ACNP) published a task force report that noted that once remission (sustained minimal symptoms over three consecutive weeks) is achieved, it can only be lost if followed by a relapse [20]. Recovery occurs after at least 4 months following remission onset without a relapse. Similar to remission, recovery is only lost if it is followed by a recurrence. It isn’t clear whether such strict definitions apply to geriatric depression. The recurrence markers, cognitive burden, and neurobiological homeostasis in depression (REMBRANDT) study’s goal of identifying risk factors and predictors of depression “relapse” following remission in recurrent LLD over 2 years is an indication of the importance of additional research in this area [21].

Over the past 15 years, there has been an increase in published reports linking adverse childhood experiences (ACEs) to later life neuropsychiatric disorders, including poor cognitive performance [22–24] and development of dementia [25], the latter finding being replicated in large databases such as the UK Biobank [26] and the Health and Retirement Study (HRS) [27, 28]. In LLD, self‐reported cumulative verbal/emotional, physical, and sexual abuse on the ACEs Questionnaire [29] was inversely associated with 1‐year change in global cognitive functioning [30]. Regarding the depression trajectory, ACEs are associated with chronic/persistent depression in adults [31–33]. However, no research has reported the relationship between ACEs and relapse following remission when adults with LLD have received treatment for recurrent depression.

Therefore, we sought to examine the relationships between recent exposure to stressful life events and ACEs with relapse following remission among older adults enrolled in evidence‐based psychopharmacological treatment for depression in the neurobiology of LLD (NBOLD) study. We hypothesized that a higher number of types of recent stressful life events and a higher total ACE score would be associated with relapse over 3 years. Given previous findings relating abuse and cognitive decline, we also hypothesized that individual ACE items, especially those assessing childhood abuse, would predict relapse and that ACEs would moderate the relationship between recent stressor exposure and relapse such that ACEs would increase the association between recent stressor exposure and relapse.

## 2. Methods

### 2.1. Subjects

All subjects were enrolled in NBOLD, a NIMH‐funded study that received ethics approval by the UConn Health Institutional Review Board (Approval Number IE‐13‐048H‐6.1). After reviewing study information, all subjects provided written informed consent to participate.

Depressed older adults were recruited from clinic referrals and newspaper advertisements. Inclusion criteria were age 65 or above, ability to read and write English, Mini‐Mental State Examination (MMSE) score 25 or greater, and meeting criteria for major depression, single episode, or recurrent. Study exclusion criteria were as follows: current or recent alcohol or drug dependence; conditions associated with brain MRI abnormalities such as hydrocephalus, benign and cancerous brain tumors, epilepsy, Parkinson’s disease, Huntington’s chorea, dementia, and demyelinating diseases; endocrine disorder other than diabetes mellitus; physical or intellectual disability that may affect completion of self‐rating instruments; established clinical diagnosis of dementia; other major DSM‐V Axis 1 psychiatric disorders; and metal or pacemaker in the body or claustrophobia that might preclude MRI. In addition, current treatment with fluoxetine was an exclusion criterion given its long washout period.

The screening and assessment procedures have been reported previously [34]. Briefly, participants were screened for depression using the Center for Epidemiologic Studies—Depression (CES‐D) scale, using a score of 16 or greater as a cut‐off for depression. Upon enrollment and completion of baseline assessments, each participant was paid $100 for their time completing all assessments, as well as a study MRI brain scan, cognitive test battery, and experimental computerized measures. The clinical assessment procedures are summarized below.

### 2.2. Baseline Assessments

Trained clinical research assistants administered the Duke Depression Evaluation Schedule (DDES) via computer‐assisted data entry to assess demographic data. The 10‐item ACE Questionnaire assessed childhood traumatic events and other forms of victimization [35]. At baseline, and as clinically indicated but at most at 12‐week intervals thereafter over a 2‐year study period, a study psychiatrist interviewed each subject to establish a clinical diagnosis of major depression and then administered the Montgomery–Asberg Depression Rating Scale (MADRS) [36].

### 2.3. Treatment Protocol

Study psychiatrists followed a treatment protocol that employed both structured and naturalistic components. All depressed subjects were offered open‐label treatment with sertraline for 12 weeks. Individuals taking antidepressants at baseline who otherwise met inclusion criteria underwent a study‐related 2‐week medication washout with weekly telephone contact to assess clinical status and provide in‐person assessments as warranted. Those who had prescribers or psychotherapists who did not wish to participate in study‐based treatment could continue medication treatment or psychotherapy outside the study. The protocol allowed for participants who could not tolerate sertraline or who failed to achieve remission with an adequate dose to either receive augmentation with bupropion or be switched to desvenlafaxine. Beyond these treatments, study psychiatrists could write a prescription for an alternate antidepressant or any medication commonly used for adjunctive treatment.

### 2.4. Statistical Approach

Our sample consisted of depressed participants whose baseline MADRS score was 15 or greater. We defined remission as achieving an MADRS score less than 8 within 2 years. Participants who subsequently worsened clinically and were rated as having a MADRS score of 15 or greater were considered to have relapsed.

All the analyses were performed using SAS 9.4. We used two types of relapse outcome measures. First, we used a dichotomous measure of relapse (yes vs. no) based on whether a participant achieved remission and experienced a relapse over 3 years. Second, time to relapse was used as a survival outcome measure. Individual ACE items were used as binary variables.

For the dichotomous measure of relapse (yes vs. no), the association with ACE items and other clinical variables of interest was first assessed using bivariate analyses, i.e., unpaired two‐sample *t*‐test for continuous variables and the chi‐square test for dichotomous variables. Multiple logistic regression models were further conducted to determine if individual ACE items, or combinations of ACE items that were statistically significant in bivariate analyses, were associated with relapse, controlling for age, sex, race, education, and baseline MADRS score.

In order to provide a more nuanced examination of the association between ACEs and relapse, survival analyses testing the relationship of ACE items with time to relapse over the study period were assessed with Kaplan–Meier plots and log‐rank tests. Those who did not achieve relapse status within 2 years after remission or who lost follow‐up within 2 years before relapse were coded as having a time‐to‐relapse of 730 days (i.e., 2 years) or the period between remission and the last time point at which they completed a study assessment, respectively. Cox proportional hazards (PHs) models were then performed to assess the associations. The adjusted hazard ratio (HR), controlling for age, sex, race, education, and baseline MADRS score, and 95% confidence limits (CLs) were reported from these models.

To assess moderation by ACE items on the association between the number of recent negative stressors and time to relapse, the interaction was added to the Cox PH models. Statistically significant interaction terms were further sliced to assess the associations at different levels of ACE items using the CONTRAST statement in the SAS PHREG procedure. Finally, for data reporting, we followed the STROBE checklist for cohort studies.

## 3. Results

Of the 141 individuals with ACE data who received initial treatment, 98 achieved remission (MADRS score < 8) within 2 years and were further followed up. During the 3‐year study period, 55 participants who achieved remission experienced a relapse (MADRS score ≥ 15), and 43 did not. Table 1 lists the demographic and clinical characteristics of those who relapsed and those who did not. Characteristics of participants with no remission data are in shown in Table S1.

**Table 1:** Demographic and clinical characteristics by relapse status.

Demographic or clinical characteristic	Nonrelapsed (*n* = 43)	Relapsed (*n* = 55)	*p*‐Value
Age (mean)	68.6 (6.7)	71.3 (6.8)	*t* (96) = −1.92, *p* = 0.058
Sex (% female)	22 (51.2%)	39 (70.9%)	*X* ^2^ (df = 1) = 4.00, *p* = 0.045
Race (% White)	39 (90.7%)	51 (92.7%)	Fisher’s exact test, *p* = 0.73
Education (mean years)	16.0 (2.4)	15.9 (2.6)	*t* (96) = 0.12, *p* = 0.90
Baseline MADRS score (mean)	20.9 (3.5)	21.4 (4.9)	*t* (95.4) = −0.59, *p* = 0.55
Number of negative stressors past year (mean)	0.84 (0.97)	1.65 (1.60)	*t* (91.0) = −3.12, *p* = 0.0024
Total ACE score (mean)	1.9 (2.0)	2.2 (2.1)	*t* (96) = −0.70, *p* = 0.48
Individual ACE items, number of yes responses (%)			
ACE1 emotional abuse	19 (44.2%)	19 (34.6%)	*X* ^2^ (df = 1) = 0.94, *p* = 0.33
ACE2 physical abuse	9 (20.9%)	14 (25.5%)	*X* ^2^ (df = 1) = 0.28, *p* = 0.60
ACE3 sexual abuse	4 (9.3%)	7 (12.7%)	Fisher’s exact test, *p* = 0.75
ACE4 emotional neglect	11 (25.6%)	25 (45.5%)	*X* ^2^ (df = 1) = 4.10, *p* = 0.043
ACE5 physical neglect	4 (9.3%)	3 (5.5%)	Fisher’s exact test, *p* = 0.70
ACE6 parental divorce	9 (20.9%)	6 (11.1%) *n* = 54	*X* ^2^ (df = 1) = 1.77, *p* = 0.18
ACE7 witness intimate partner violence	5 (11.9%) (*n* = 42)	7 (12.7%)	*X* ^2^ (df = 1) = 0.015, *p* = 0.90
ACE8 family substance misuse	8 (18.6%)	14 (25.9%) *n* = 54	*X* ^2^ (df = 1) = 0.73, *p* = 0.39
ACE9 family mental illness	11 (25.6%)	24 (43.6%)	*X* ^2^ (df = 1) = 3.43, *p* = 0.064
ACE10 family member incarcerated	2 (4.7%)	2 (3.6%)	Fisher’s exact test, *p* = 1.00
ACE4 or 9 endorsed, *N* (%)	17 (39.5%)	36 (65.5%)	*X* ^2^ (df = 1) = 6.53, *p* = 0.011
Sum of ACE4 and 9, mean (SD)	0.51 (0.70)	0.89 (0.76)	*t* (96) = −2.53, *p* = 0.013

As shown in Table 1, in bivariate analyses, only one ACE item (ACE4, emotional neglect) was associated with relapse. ACE item 9 (household member with mental illness) approached significance (*p* = 0.06). We then created two variables combining ACE4 and ACE9: (ACE4 + ACE9) is the sum of ACE4 and ACE9, and (ACE4 or ACE9) is a binary variable with “neither item endorsed” coded as “NO” compared to “at least 1 item endorsed” as “YES.” Additional bivariate analyses showed that these two variables, combining ACE4 and ACE9, were significantly related to relapse. In contrast to our a priori hypothesis that childhood abuse would be associated with relapse, no ACE item related to abuse (ACE1, 2, or 3) was associated with relapse.

In subsequent logistic regression models, controlling age, sex, race, education, and baseline MADRS score, neither emotional neglect (ACE4) nor family mental illness (ACE9) had a statistically significant association with relapse (odds ratio [OR] with 95% confidence interval [CI] = 2.26 [0.90, 5.63], *p* = 0.082 for ACE4; OR [95% CI] = 1.92 [0.77, 4.79], *p* = 0.16 for ACE9). The dichotomous and continuous variables combining items 4 and 9 were significantly associated with relapse (OR with 95% CI = 2.64 [1.11, 6.27], *p* = 0.028 for either ACE4 or ACE9 endorsed; OR [95% CI] = 1.84 [1.01, 3.36], *p* = 0.046 for ACE4 + ACE9).

### 3.1. Survival Analyses

In analyses using time‐to‐relapse as the outcome, Kaplan–Meier analysis showed that a “yes” response to ACE4 (emotional neglect) was associated with shorter time to relapse (median [95% CL]: 265 days [175, 465]) compared with a “no” response (median [95% CL]: 651 days [343, ∞]), (*p* value = 0.027, Figure 1a).

**Figure 1:**
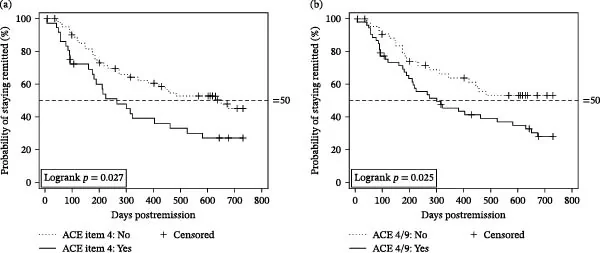
Time‐to‐relapse Kaplan–Meier curves. (a) ACE4 (emotional neglect) and time to relapse. (b) ACE4 (emotional neglect) or ACE9 (family mental illness) and time to relapse.

For ACE9 (family mental illness), the Kaplan–Meier analysis showed that median time‐to‐relapse was 273 days (175, 651) for ACE9 yes response vs. 483 days (315, ∞) for the no response, although this result was not statistically significant (*p* = 0.084).

For the binary version of combined ACE4 and ACE9, the Kaplan–Meier analysis revealed that 25th percentile time‐to‐relapse was 120 days (77, 197) for the yes response vs. 189 days (126, 420) for the no response, also a significant association (*p* = 0.025) (See Figure 1b).

The Cox PH model controlling for age, sex, race, education, and baseline MADRS score showed that the risk of relapse in participants reporting ACE4 increased by 63% (HR [95% CL] = 1.63 [0.94, 2.82], *p* = 0.082).

### 3.2. Recent Stressor Analyses

Cox PHs models revealed that a higher number of recent negative stressors was associated with an increased risk of relapse (HR [95% CL]:1.30 [1.06, 1.57]). In analyses examining whether ACE4 and/or ACE9 moderate this relationship, as shown in Table 2, the interaction of ACE4 by number of recent stressors was statistically significant (*p* = 0.027), indicating moderation by ACE4. In participants not endorsing ACE4, the risk of relapse increased by 50% with every unit increase of recent stressor exposure (HR [95% CL]: 1.50 [1.18, 1.87]), while the association between negative stressors and the risk of relapse was not observed in participants who endorsed ACE4 (HR [95% CL]: 0.93 [0.64, 1.30]).

**Table 2:** Cox proportional hazards model for moderation effect of ACE item 4 on the association between number of recent stressors and time to relapse.

Model variable	HR (95% CL)			*p*‐Value
ACE4 (emotional neglect)	—	—	—	0.071
Number of negative stressors	—	—	—	0.0005
ACE4 × number of negative stressors				0.027
ACE4 = no	1.50	1.18	1.87	0.0005
ACE4 = yes	0.93	0.64	1.30	0.68
Female (reference category: male)	1.67	0.93	3.13	0.095
Age	1.03	0.99	1.07	0.12
Other race (reference category: White)	0.75	0.22	1.95	0.60
Education level	0.99	0.89	1.10	0.81
Baseline MADRS	1.03	0.96	1.10	0.40

Neither ACE9 nor the binary version of combined ACE4 and ACE9 showed a similar moderation effect (the *p* values of interaction terms are 0.48 and 0.16, respectively).

## 4. Discussion

The main finding of this study is that exposure to emotional neglect in childhood and stressors later in life both were associated with relapse of LLD, and a history of childhood neglect moderated the relationship between recent stressor exposure and relapse. Unexpectedly, adults with LLD and a history of childhood neglect were less likely to relapse in the context of recent life stressors than those not reporting a history of childhood neglect.

The relationship between adversity/stressor exposure and recurrent depression in older adulthood thus includes the potentially developmentally adverse distal effects of interpersonal stressors in childhood as well as the effects of contemporaneous stressors in older adulthood. These effects also were independent of and not accounted for by the severity of depression at the outset of treatment, suggesting that they apply to the full range of older adults who seek treatment for depression and not just to those with the most severe depressive symptoms.

The association between the number of negative life stressors experienced and depression relapse is consistent with previous studies showing a relationship between exposure to negative life events in older adulthood and relapse following successful treatment for depression [17, 18]. The current findings are the first to show that late‐life stressor exposure increases the risk of depression even when remission was achieved with evidence‐based psychopharmacological treatment for depression. This implies that ongoing clinical monitoring of depression symptoms is particularly important following successful treatment when older adults are affected by negative life stressors in order to provide timely and targeted interventions to facilitate effective coping with stressors and ameliorate or prevent depression recurrence.

The finding that only two of the 10 ACEs items were associated with depression relapse adds nuance to prior research results linking ACEs to depression and related internalizing and stressor‐related disorders among older adults [20–22; 29–31]. In contrast with prior findings [37–39], the cumulative burden of ACEs and exposure to the abuse‐related types of ACEs were not associated with relapse.

However, ACEs involving psychological neglect and a household member affected by a mental illness such as depression each were associated with an increased risk of relapse. In addition, a history of childhood neglect was associated with a shorter time to depression relapse. The findings regarding childhood neglect are consistent with and extend the robust research evidence base linking psychological neglect with depression in adolescence and adulthood [38, 40–42]. The finding linking familial mental illness to relapse is consistent with the evidence of intergenerational transmission of depression in childhood and young adulthood, extending this relationship to depression in older adults [43–47].

Unexpectedly, adults with LLD who reported a history of childhood neglect did not show a relationship between recent negative life stressors and depression relapse, in contrast to those with no history of neglect, for whom there was a strong adverse effect of negative life stressors on the risk of relapse. Childhood neglect has been found to be associated with blunted emotion regulation, impairment in the development of executive function, and detachment in close relationships in childhood and adolescence [48, 49]. Although research on the association of childhood neglect with adult emotion regulation is still preliminary, difficulties with emotion awareness and clarity have been identified as potential sequelae of childhood neglect [50]. Thus, childhood neglect may lead to susceptibility to depression regardless of the effects of subsequent stressors, which may be due to a combination of increased risk of chronic dysphoria and relative insensitivity to negative life stressors. Alternately, such apparent hardiness may reflect vulnerability (i.e., susceptibility to relapse even when not exposed to current negative life stressors) rather than resilience to psychopathology. Both possibilities warrant further research.

Limitations of the study include a relatively small sample size. Future studies with larger samples of older depressed adults are needed to replicate the findings and to determine if ACEs other than neglect and a familial history of mental illness are associated with relapse and moderate the association between recent cumulative stressor exposure and LLD relapse. Another limitation was that the relationship of other childhood adversities, other than neglect and family mental illness, with relapse was not tested on a multivariate. This was not done in order to reduce the risk of artifactual statistical findings by limiting multivariate analyses to ACEs that were associated with relapse on a bivariate basis but should be done in future research. Additionally, the ACEs questionnaire was used to provide results comparable to those of the many other studies in which the measure has been widely used, but it does not assess many potentially traumatic adversities of childhood nor the developmental timing, severity, chronicity, or other important features of adversity (e.g., relationship to maltreatment perpetrators) that are important to understand in both research and clinical applications of ACEs.

## 5. Conclusions

These findings highlight the importance of assessing early life trauma, especially neglect, as part of the evaluation of LLD. Further studies are warranted to examine the effects of exposure to traumatic stressors in childhood on stress experienced during adulthood (i.e., revictimization), including stressors occurring later in life.

## Funding

This work was supported by the U.S. National Institutes of Health (Grants R01MH108578 and P30AG067988).

## Conflicts of Interest

The authors declare no conflicts of interest.

## Supporting Information

Additional supporting information can be found online in the Supporting Information section.

## Supporting information


**Supporting Information** Table S1: Summarized demographic and clinical characteristics of 43 individuals who were excluded from the analysis for relapse due to non‐remission and therefore a lack of a valid depression relapse status.

## Data Availability

The data that support the findings of this study are openly available in the NIMH Data Archive at https://nda.nih.gov.
