# Supplementary material for: Childhood Trauma, Recent Stressors, and Risk of Depression Relapse in Late‐Life Depression
Source: Depress Anxiety. 2026 Oct 8;2026:1455337. doi: 10.1155/da/1455337 (PMC13647104; doi:10.1155/da/1455337)
Supplement: Supplementary file 1 [file DA-2026-1455337-s001.docx]

**Supplementary Information**

**Table S1. Demographic and clinical characteristics of those individuals who failed to achieve remission within two years and thus had no valid relapse status (n=43)**

| Age, mean | 71.2 (6.6) |
| --- | --- |
| Sex, % female | 29 (67.4%) |
| Race, % White | 35 (81.4%) |
| Education, mean years | 15.6 (2.4) |
| Baseline MADRS score, mean | 21.4 (5.0) |
| Number of negative stressors past year, mean | 1.88 (1.60) |
| Total ACE score, mean | 2.5 (2.2) |
| Individual ACE items, number of yes responses (%) |  |
| ACE1 Emotional abuse | 20 (46.5%) |
| ACE2 Physical abuse | 12 (27.9%) |
| ACE3 Sexual abuse | 12 (27.9%) |
| ACE4 Emotional neglect | 15 (34.9%) |
| ACE5 Physical neglect | 5 (11.6%) |
| ACE6 Parental divorce | 11 (25.6%) |
| ACE7 Witness intimate partner violence | 4 (9.3%) |
| ACE8 Family substance misuse | 11 (25.6%) |
| ACE9 Family mental Illness | 15 (34.9%) |
| ACE10 Family member incarcerated | 2 (4.7%) |
|  |  |
| ACE4 or 9 endorsed, N (%) | 22 (51.2%) |
| Sum of ACE4 and 9, Mean (SD) | 0.70 (0.77) |

Note: We had 141 individuals with ACE data who received initial treatment. 43 IDs (=141-98) did not have a valid depression relapse status and were therefore excluded in the analysis for relapse outcomes. Among these 43 IDs, 15 IDs did not achieve remission within 2 years; 24 IDs had less than 2-year follow-up from baseline and their remission status could not be determined; 4 IDs achieved remission at last timepoint and had no further follow-up for relapse status.
